# Associations between Multiple Food Consumption Frequencies and the Incidence of Cardiovascular Disease in High Cardiac Risk Subjects

**DOI:** 10.31083/j.rcm2511412

**Published:** 2024-11-20

**Authors:** Xiaohui Xu, Shiyun Hu, Sijie Shen, Fang Ding, Jianlin Shao, Xiafen Shen, Tianxu Chen, Xiaoling Xu, Jing Yan, Yin Zhu, Qiang Cai, Wei Yu

**Affiliations:** ^1^Zhejiang Provincial Center for Cardiovascular Disease Control and Prevention, Zhejiang Hospital, 310013 Hangzhou, Zhejiang, China; ^2^Department of Food and Agricultural Technology, Yangtze Delta Region Institute of Tsinghua University, 314006 Jiaxing, Zhejiang, China

**Keywords:** dietary, cardiovascular disease, LASSO, Cox frailty models, nomogram

## Abstract

**Background:**

Dietary choices are inextricably linked to the incidence of cardiovascular disease (CVD), whereas an optimal dietary pattern to minimize CVD morbidity in high-risk subjects remains challenging.

**Methods:**

We comprehensively assessed the relationship between food consumption frequencies and CVD in 28,979 high-risk subjects. The outcome was defined as the composite of the incidence of major CVD events, including coronary heart disease and stroke. Risk factors associated with CVD were screened through a shrinkage approach, specifically least absolute shrinkage and selection operator (LASSO) regression. Hazard ratios (HRs) for various dietary consumption frequencies were assessed using multivariable Cox frailty models with random intercepts.

**Results:**

Increased egg and seafood consumption were associated with a lower risk of CVD (daily vs little, HR 1.70, 95% confidence interval, CI: 0.79–3.64, *p*_trend_ = 0.0073 and HR 1.86, 95% CI: 1.24–2.81, *p*_trend_ = 0.024, respectively). 6 non-food (age, sex, smoke, location, heart ratio, and systolic blood pressure) and 3 food (fruit, egg, and seafood) related risk factors were included in the nomogram to predict 3 and 5-year incidence of CVD. The concordance indexes of the training and validation cohorts were 0.733 (95% CI: 0.725–0.741) and 0.705 (95% CI: 0.693–0.717), respectively. The nomogram was validated using the calibration and time-dependent receiver operating characteristic curves, demonstrating respectable accuracy and discrimination.

**Conclusions:**

Guided by the concept of “food as medicine”, this nomogram could provide dietary guidance and prognostic prediction for high cardiac risk subjects in CVD prevention.

## 1. Introduction

Cardiovascular disease (CVD) is one of the leading causes of death globally, particularly in developing countries, accounting for over 75% of CVD-related mortality [1, 2]. In 2019, there were approximately 330 million CVD patients and over 4 million CVD-related deaths in China, representing 43% of all deaths in the country [3, 4]. Early intervention is crucial for preventing and managing CVD [5]. Epidemiological studies have demonstrated that healthy diets are associated with a reduced risk of CVD incidence in individuals without prior CVD [6]. Generally, diets low in added sugars, salt, animal-source foods, and refined grains and high in vegetables, fruits, fish, beans, and whole grains are defined as high-quality diets [7].

Many studies [8, 9] have concentrated on the relationship between specific nutrients in various foods and CVD incidence. Some epidemiological studies [10, 11] have directly linked dietary patterns to CVD events in different populations. For instance, the Mediterranean diet has been shown to reduce the risk of CVD in randomized clinical trials (RCTs) [12]; a Western diet high in ultra-processed foods is associated with a higher risk of coronary heart disease (CHD) [13]; a vegetarian diet can reduce CVD and CHD mortality by 40% [14]; and the dietary approach to stop hypertension (DASH) diet has been proven to significantly lower blood pressure in hypertensive individuals in the United States [15]. Additionally, some investigations showed that incorporating certain foods into one’s diet can be highly beneficial for cardiovascular health. For example, whole grains significantly reduce the risk of CHD, with each additional serving per day decreasing the risk by 7% (hazard ratio, HR 0.93, 95% confidence interval, CI: 0.90–0.95) [16]. Omega-3 fatty acids in fish contribute to heart health by reducing inflammation and improving lipid profiles [17]. Daily intake of fresh vegetables and fruits is linked to lower systolic blood pressure and blood glucose levels [18]. Legumes, such as beans, also play a protective role, with regular consumers experiencing an 11% lower risk of CVD compared to those who consume legumes infrequently [19]. Moderate egg consumption, up to one egg per day, has not been found to increase heart disease risk significantly, suggesting it can be part of a heart-healthy diet [20]. Conversely, certain dietary choices can negatively impact cardiovascular health. High intake of refined grains, such as white rice, white flour, and white bread, is associated with an increased risk of heart disease and mortality, although refined white rice does not appear to increase these risks specifically [21]. Unprocessed red meat, such as beef, pork, and lamb, is linked to a higher risk of CVD, highlighting the need for moderation in consumption [20]. Preserved vegetables, unlike their fresh counterparts, are marginally associated with higher CVD mortality, indicating that not all vegetable sources provide the same health benefits [22]. Furthermore, a higher intake of total dairy and milk has been positively associated with an increased risk of stroke and cardiovascular mortality, suggesting potential risks in excessive consumption [19]. Studying whole food patterns allows for a more comprehensive understanding of their effects on health, and studying the specific effects of individual foods can optimize dietary structure for cardiovascular health. However, few studies appraised the impact of multiple food consumption frequencies on CVD incidence. Furthermore, no nomogram is currently available that links multiple food consumption frequencies to CVD events in high-risk cardiac individuals.

To address these gaps, we examined the associations between multiple food consumption frequencies and CVD incidence based on the perspective cohort from the China Health Evaluation and Risk Reduction through Nationwide Teamwork (ChinaHEART) project, where we analyzed various food consumption frequencies, including rice, wheat, grains, poultry, meat, seafood, eggs, vegetables, pickled vegetables, fresh fruit, beans, and dairy using the least absolute shrinkage and selection operator (LASSO) and multivariable Cox frailty models. For the application in a convenient manner, we constructed and validated a nomogram using the statistically significant CVD-related individual characteristics and food consumption frequencies.

## 2. Materials and Methods 

### 2.1 Data Sources

The dataset was obtained from the baseline of the ChinaHEART study with detailed information previously published elsewhere [23]. In brief, 28,979 high-risk CVD subjects aged 35–75 years from the center of Zhejiang province were selected for the analysis from 199,571 baseline subjects who completed questionnaires between September 2014 and March 2021. High-risk cardiac participants were identified based on World Health Organization (WHO) guidelines, which include a more than 20% CVD risk in 10 years, a history of CVD event, high blood pressure, and dyslipidemia [24]. Physical examinations, sociodemographic surveys, and laboratory tests were conducted as per previous studies [25, 26]. In brief, trained medical staff collected the CVD-related data of participants using a questionnaire designed by Fuwai Hospital. Ethical approval was granted by the Zhejiang Hospital Medical Ethics Committee (2019 No.27K). All participants provided informed consent in writing, either by thumbprint or signature. The flow chart for selecting high-risk cardiac subjects is shown in **Supplementary Fig. 1**.

### 2.2 Dietary Assessment

The dietary intake of high-risk cardiac participants were recorded through questionnaires, which were self-reported as food consumption frequency: 1 = Daily; 2 = 4–6 days a week; 3 = 1–3 days a week; 4 = 1–3 days a month; 5 = None or little [23]. The study covered 12 food items, including rice, wheat, grains, poultry, meat, seafood, eggs, vegetables, pickled vegetables, fresh fruit, beans, and dairy.

### 2.3 Covariates and Outcomes

Baseline covariates included in the following analysis were age, sex, smoking status, educational level, urban or rural location, drinking status, body mass index (BMI), systolic blood pressure (SBP), diastolic blood pressure (DBP), heart rate (hr), high-density lipoprotein (HDL), low-density lipoprotein (LDL), total cholesterol (TC), triglyceride (TG). BMI was calculated by dividing weight in kilograms by height in meters squared. Missing values were imputed using multiple imputations [27]. CVD outcome was defined as the composite of the occurrence of stroke and CHD.

### 2.4 LASSO Regression and Cox Regression for Multivariate Prognostic Model

LASSO regression was employed for covariate selections and implemented using the “glmnet” R (version 4.0.3, R Core Team, Vienna, Austria) package with 10-fold cross-validation to determine optimal penalty parameters. After screening all variables using LASSO regression, univariate and multivariate Cox regression models were used to evaluate the statistically significant non-food risk factors (covariates). Multivariable Cox frailty models were constructed based on food consumption frequency, statistically significant covariates. The selected significant dietary and non-dietary parameters were used to construct a predictive model to minimize model errors. Categorical variables included gender, smoking status, educational level, and drinking status.

### 2.5 Construction and Validation of the Nomogram

A nomogram was constructed using selected variables to predict CVD incidence based on food consumption frequency. The “rms” R package was used to plot the nomogram for predicting CVD incidence at 3 and 5-years. Both training and validation cohorts were used to construct and validate the nomograms. The Kaplan-Meier method was used to conduct survival analysis. The prediction ability of the nomogram was evaluated using bootstrap self-sampling and concordance index (C-index). The consistency index between the actual observations and predicted probabilities estimated the accuracy of the nomogram. The prediction accuracy of the continuous variable, known as the risk score, was assessed using a time-dependent receiver operator characteristic (ROC) curve. Decision curve analysis (DCA) was used to determine a net-benefit threshold of prediction.

### 2.6 Statistical Analysis

After identifying the high-risk population, the dataset was randomly divided into training and validation cohorts at a ratio of approximately 2:1. Missing data were handled using multiple interpolations. Continuous variables with normal and non-normal distribution were summarized using means with standard deviations and medians with interquartile ranges (IQRs), respectively. Comparisons among clinical characteristics were conducted using *t*-tests, Mann-Whitney U tests, or Chi-square tests for categorical variables appropriately. Following LASSO regression, all non-food-related covariates underwent separate multivariable regression to identify significant variables. The significant results were then incorporated with the food-related variables into the subsequent multivariable Cox frailty models. Specifically, model 1 included age, sex, and center as random effects, while model 2 included age, sex, alcohol consumption, location, smoking status, educational level, BMI, LDL, HDL, TC, hr, SBP, DBP, and center as random effects [28]. Multivariable Cox frailty models automatically adjusted for different center clustering. HR and 95% CI were estimated for each factor. Statistical analyses and figures were generated using R (Version 4.2.1, R Foundation for Statistical Computing, Vienna, Austria). The 3 and 5-year predicted probability of CVD were calculated for each high cardiac risk subject using the Cox regression model underlying the nomogram.

## 3. Results

### 3.1 Clinical Features and Characteristics

**Fig. 1.**
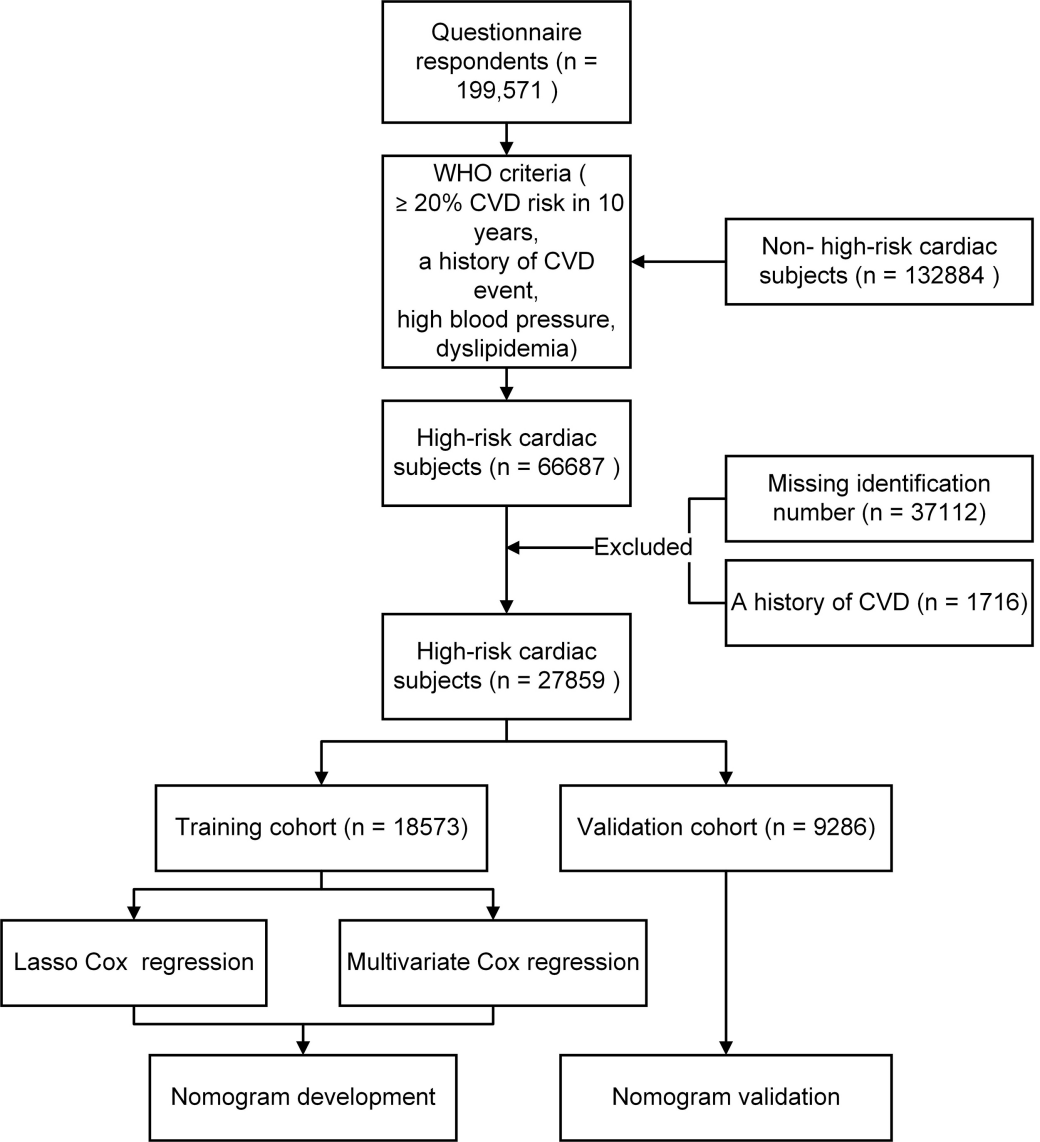
**Flowchart for Selecting high-risk cardiac subjects**. Abbreviation: CVD, cardiovascular disease; WHO, World Health Organization.

The study flowchart is shown in Fig. 1. The training cohort comprised 19,320 participants with high cardiac risk (mean age 58.57 years; 46.72% male), which were enrolled between September 2014 and March 2021. Two-thirds of them were allocated to the training cohort, and one-third to the validation cohort (Table 1). Over a median follow-up of 5.94 years, CVD incidences were 4.05% (n = 783) in the training and 4.26% (n = 411) in the validation cohorts. Since these continuous variables do not follow a normal distribution, medians with interquartile ranges were used in this study. Participants who developed CVD were more likely to be male. Characteristics related to food consumption frequencies are detailed in **Supplementary Table 1**.

**Table 1.** **Characteristics of high cardiac risk subjects in the training and validation cohorts**.

		Training cohort (No CVD)	Training cohort (CVD)	Validation cohort (No CVD)	Validation cohort (CVD)	*p*
n		18,537	783	9248	411	
Age (median [IQR])		59.00 [51.00, 65.00]	66.00 [60.00, 70.00]	59.00 [52.00, 65.00]	66.00 [59.50, 71.00]	<0.001
Sex = Female (%)		10,006 (53.98)	326 (41.63)	4916 (53.16)	193 (46.96)	<0.001
Education (%)						<0.001
	Post-secondary school	1046 (5.64)	32 (4.09)	527 (5.70)	18 (4.38)	
	Pre-secondary school	4452 (24.02)	230 (29.37)	2154 (23.29)	136 (33.09)	
	Secondary school	13,039 (70.34)	521 (66.54)	6567 (71.01)	257 (62.53)	
BMI (median [IQR])		24.92 [22.86, 27.11]	24.96 [22.80, 27.06]	24.87 [22.83, 27.06]	24.73 [22.77, 27.35]	0.885
SBP (median [IQR])		161.50 [142.50, 170.00]	165.00 [153.50, 175.50]	162.00 [143.50, 170.50]	164.50 [152.00, 175.00]	<0.001
DBP (median [IQR])		88.00 [80.00, 97.00]	88.50 [80.00, 98.00]	88.50 [80.00, 97.50]	89.00 [80.50, 97.00]	0.144
hr (median [IQR])		75.50 [69.00, 83.50]	77.00 [69.50, 85.00]	76.00 [69.50, 83.50]	77.00 [70.00, 85.50]	0.023
TC (median [IQR])		5.10 [4.30, 6.24]	5.10 [4.24, 5.93]	5.09 [4.26, 6.21]	5.05 [4.31, 6.05]	0.149
HDL (median [IQR])		1.43 [1.11, 1.77]	1.44 [1.14, 1.76]	1.42 [1.11, 1.76]	1.45 [1.13, 1.81]	0.455
TG (median [IQR])		1.71 [1.15, 2.60]	1.71 [1.11, 2.60]	1.70 [1.13, 2.59]	1.76 [1.24, 2.64]	0.543
LDL (median [IQR])		2.89 [2.14, 3.66]	2.79 [2.12, 3.40]	2.87 [2.13, 3.59]	2.84 [2.08, 3.56]	0.019
Location = Urban (%)		4045 (21.82)	215 (27.46)	1958 (21.17)	112 (27.25)	<0.001
Smoke = 1 (%)		3789 (20.44)	215 (27.46)	1922 (20.78)	109 (26.52)	<0.001
Alcohol = 1 (%)		4062 (21.91)	190 (24.27)	2125 (22.98)	90 (21.90)	0.12

Abbreviations: CVD, cardiovascular disease; BMI, body mass index; SBP, systolic blood pressure; DBP, diastolic blood pressure; hr, heart ratio; TC, total cholesterol; TG, triglyceride; HDL, high-density lipoprotein; LDL, low-density lipoprotein; IQR, interquartile range.

### 3.2 LASSO Regression and Cox Regression for Variable Selection

**Fig. 2.**
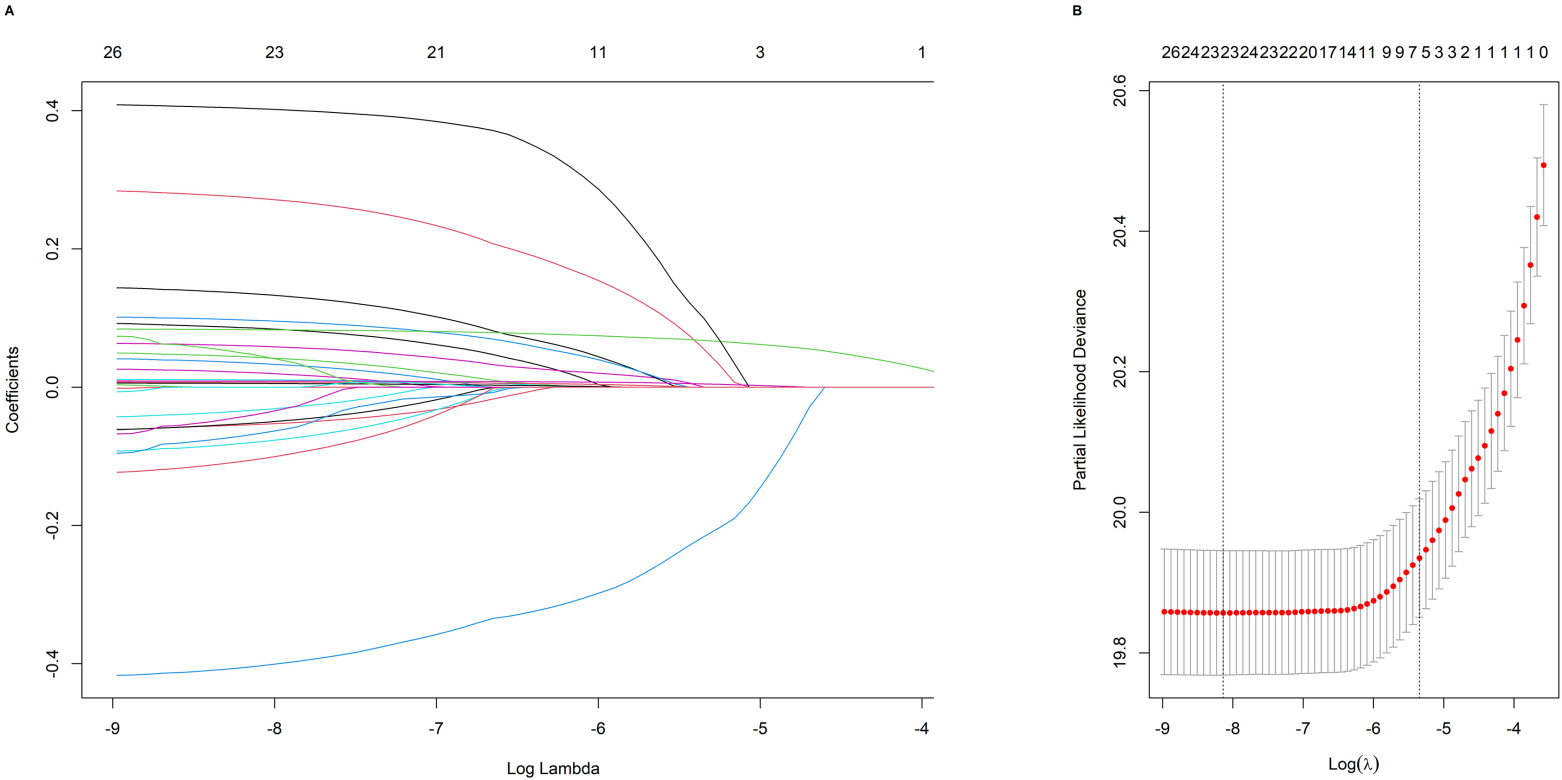
**Analysis of LASSO regression models for selecting factors**. (A) LASSO coefficients for 26 candidate variables. (B) The optimal penalty coefficient (λ) was found using cross-validation and the minimum criterion. The cross-validated error within one standard error (1-s.e.) of the minimal error is shown on the right, while the minimum error is shown on the left. Abbreviations: LASSO, Least absolute shrinkage and selection operator.

LASSO-penalized Cox analyses were applied to the training cohort to identify CVD risk factors (Fig. 2A). The LASSO method for selecting independent variables is illustrated in Fig. 2. Thirteen risk factors were selected for population characteristics and sociological characteristics were screened, including age, sex, alcohol, location, smoking status, education, LDL, HDL, TC, hr, DBP, SBP, and BMI. The 11 dietary factors included were dairy, beans, fruit, pickles, eggs, seafood, poultry, meat, grain, wheat, and rice. The coefficient of each variable at the minimum error is shown in **Supplementary Table 2**. According to **Supplementary Table 3**, the non-food variables that are statistically significant in the multivariate Cox regression model are sex (HR 0.66, 95% CI: 0.55–0.80), age (HR 1.09, 95% CI: 1.08–1.10), HR (HR 1.01, 95% CI: 1.00–1.01), SBP (HR 1.01, 95% CI: 1.00–1.01), smoke (HR 1.36, 95% CI: 1.13–1.64), and location (HR 1.57, 95% CI: 1.07–2.29).

### 3.3 Cardiovascular Events Associated with Food Consumption

**Fig. 3.**
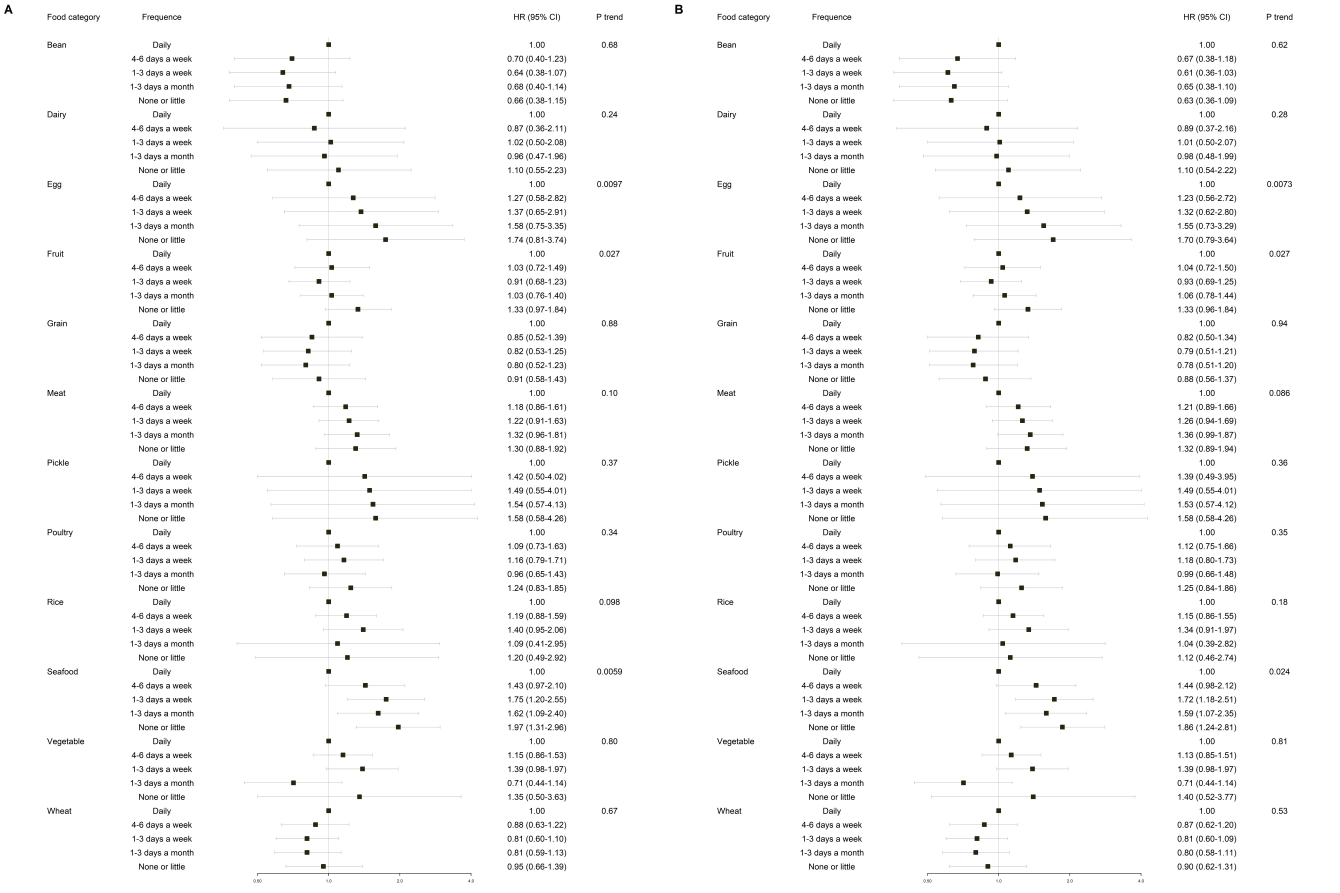
**Forest plots of adjusted hazard ratios for associations between food consumption and CVD events in the training cohort**. (A) model 1: adjusted for age, sex, and center as a random effect. (B) model 2: adjusted for age, sex, alcohol, location, smoke, education, BMI, LDL, HDL, TC, hr, DBP, SBP, and center was also included as a random effect. Confidence intervals are 95%. Abbreviations: CVD, cardiovascular disease; BMI, body mass index; DBP, diastolic blood pressure; SBP, systolic blood pressure; hr, heart ratio; TC, total cholesterol; LDL, low-density lipoprotein; HDL, high-density lipoprotein.

The forest plot of adjusted hazard ratios is shown in Fig. 3. After adjustment in model 1, minimal seafood consumption significantly increased the risk of CVD events compared to daily consumption (HR 1.86, 95% CI: 1.24–2.81; *p*_trend_ = 0.024); Similarly, little egg consumption compared to daily consumption was associated with an increased risk of CVD incidence (HR 1.70, 95% CI: 0.79–3.64; *p*_trend_ = 0.0073); Compared with daily intake, lower consumption of fruit (1–3 days a week) was associated with a reduced CVD risk (HR 0.93, 95% CI: 0.69–1.25; *p*_trend_ = 0.027). These results remained consistent after further adjustment in model 2. Besides, model 2 revealed that little consumption of rice (HR 1.12, 95% CI: 0.46–2.74; *p*_trend_ = 0.18), meat (HR 1.32, 95% CI: 0.89–1.94; *p*_trend_ = 0.086), poultry (HR 1.25, 95% CI: 0.84–1.86; *p*_trend_ = 0.35) and pickle (HR 1.58, 95% CI: 0.58–4.26; *p*_trend_ = 0.36) were associated with an increased CVD risk compared to daily consumption. However, these associations were not statistically significant. Detailed information on HRs for each dietary frequency is shown in **Supplementary Table 4**.

### 3.4 Construction of the Nomogram

**Fig. 4.**
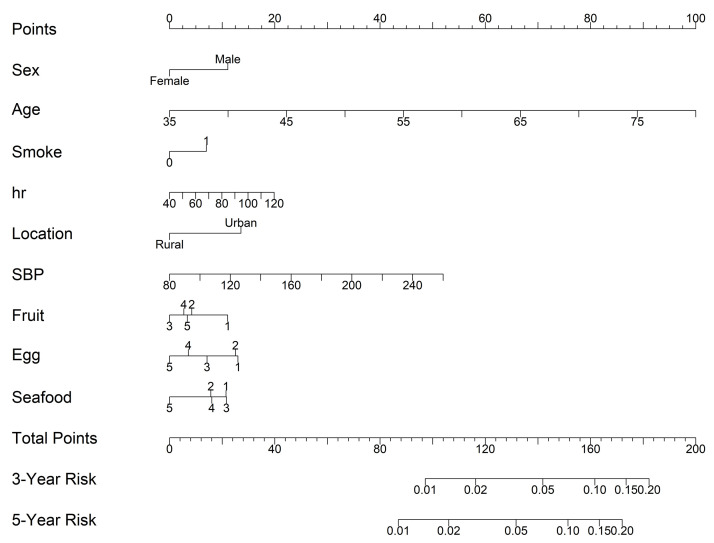
**A nomogram for predicting the incidence of CVD and associated food consumption in high cardiac risk subjects**. Food consumption frequency: 1 = Daily; 2 = 4–6 days a week; 3 = 1–3 days a week; 4 = 1–3 days a month; and 5 = None or little. Notes: By drawing a line upward from the point reference line, the value for each predictor is determined. Abbreviations: CVD, cardiovascular disease; hr, heart ratio; SBP, systolic blood pressure.

9 statistically significant factors were incorporated into the nomogram models based on the Cox regression analysis: age, sex, smoke, location, hr, SBP, fruit, egg, and seafood consumption. The nomogram was constructed to predict the 3 and 5-year risk of CVD in the training cohort (Fig. 4). Age, with a score of 100, has the greatest impact on prognosis, followed by SBP and hr. The probability of CVD incidence was determined simply by looking at the points corresponding to each variable on the survival scale. Each variable score was added up, and the risk measures were computed. In this case, the results could assist in formulating individualized dietary management plans for the patients.

### 3.5 Validation of the Nomogram

**Fig. 5.**
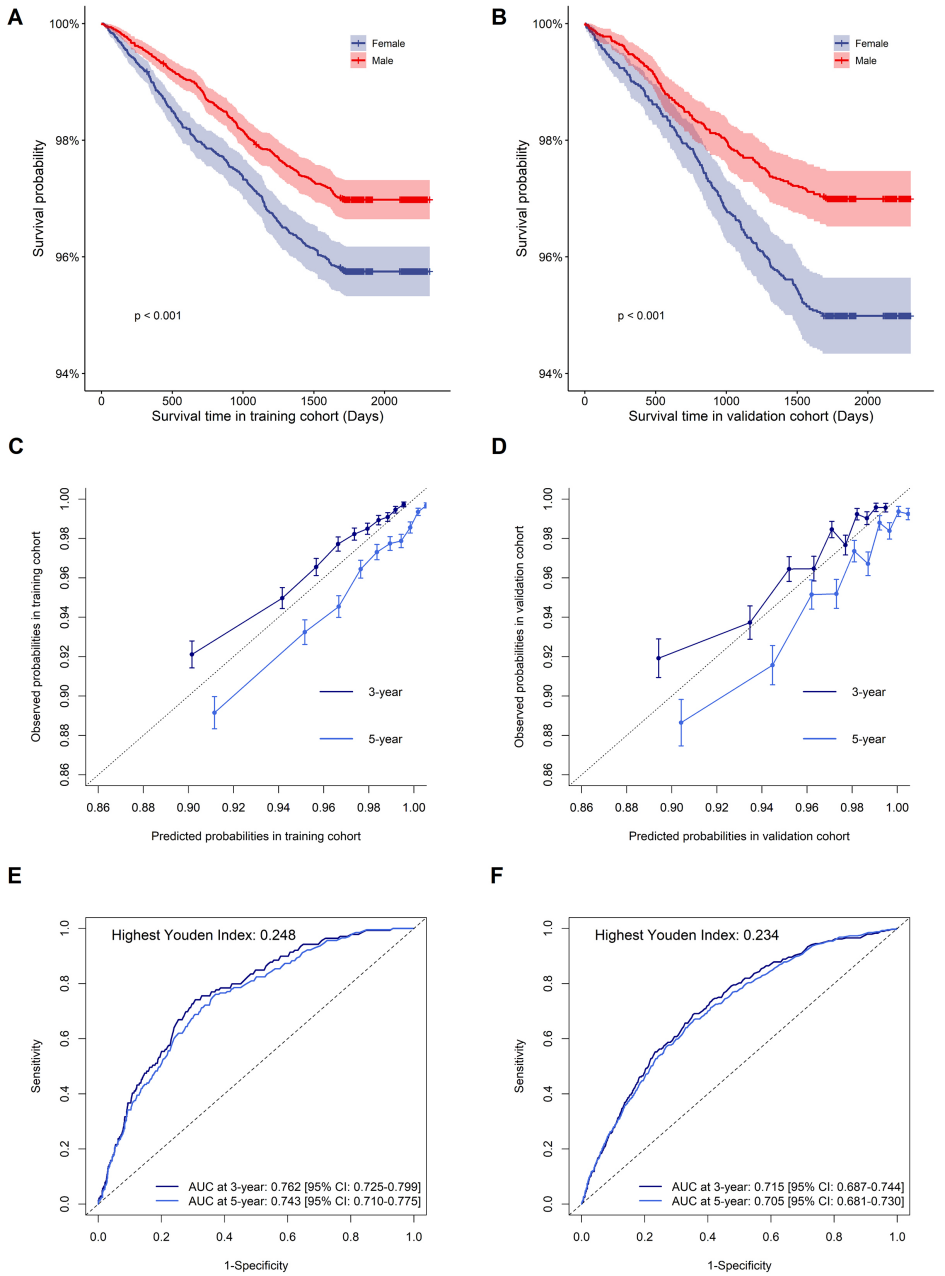
**The survival curve, calibration plot, and time-dependent ROC curve of the nomogram in the training and validation cohorts**. (A,B) Kaplan-Meier survival for the incidence of CVD between males and females in the training and validation cohorts. (C,D) Calibration plot of 3 and 5-year CVD risk prediction in the training and validation cohorts. (E,F) Time-dependent ROC curves in the training and validation cohorts. Abbreviations: CVD, cardiovascular disease; ROC, receiver operator characteristic; AUC, area under the curve. Youden index = sensitivity + specificity – 1.

The nomogram’s performance was evaluated, yielding a C-index of 0.733 (95% CI: 0.725–0.741) in the training cohort and 0.705 (95% CI: 0.693–0.717) in the validation cohort, respectively. Kaplan-Meier survival curves for training and validation cohorts according to sex are shown in Fig. 5A,B, indicating higher CVD risk in males compared to females. Using bootstrapping with 1000 resamples, the nomogram calibration plots are displayed in Fig. 5C,D, indicating that the model’s predicted probabilities closely matched the observed probabilities in both cohorts. The area under the curve (AUC) values at 3 and 5-years in the training cohort were 0.762 [95% CI: 0.725–0.799] and 0.743 [95% CI: 0.710–0.775], respectively; and the AUC values at 3 and 5-years in the validation cohort were 0.715 [95% CI: 0687–0.744] and 0.705 [95% CI: 0.681–0.730], respectively (Fig. 5E,F). The DCA curve of the nomogram was shown in **Supplementary Fig. 1**, indicating a possible clinical benefit from the nonogram which was developed.

## 4. Discussion

This study investigated the relationship between food consumption frequencies and CVD in high-risk subjects, highlighting that increased consumption of eggs and seafood is associated with a lower risk of CVD. The nomogram was developed by integrating statistically significant variables such as age, sex, smoking, location, hr, SBP, and specific dietary components provide a useful predictive tool for assessing CVD risk. Fruit consumption demonstrated a beneficial effect, showing a lower CVD risk with moderate intake (1–3 days per week) compared to little consumption. This finding is consistent with previous studies, which observed limited additional benefit beyond this frequency [29, 30]. This effect may be attributed to the presence of antioxidants, dietary fiber, vitamins, and minerals in fruits, which contribute to improved cardiovascular health by reducing oxidative stress, inflammation, and improving endothelial function [31]. Similarly, seafood consumption, rich in long-chain n-3 polyunsaturated fatty acids like docosahexaenoic acid, was associated with reduced CVD risk, supported by its favorable effects on lipid profiles [32, 33, 34]. In contrast, the relationship between egg consumption and CVD risk is complex and varies across populations. While some studies suggest a protective effect, others indicate potential risks associated with higher intake levels [35, 36, 37]. Our study reinforces the need for cautious interpretation regarding the impact of egg consumption impact on CVD, given the inconsistent findings across different populations and health conditions [38, 39]. Despite genetically predicted associations between higher dairy consumption and favorable lipid profiles, this study did not find a significant link to reduced CVD outcomes, suggesting further investigation is warranted [40]. It is undeniable that some components in ultra-processed foods may contribute to increased blood pressure and lipid levels [41, 42]. Interestingly, higher pickle intake showed a modest reduction in major CVD events, potentially attributed to probiotics present in some pickles, although this requires validation through extended follow-up and detailed processing method considerations [43]. Vegetable consumption, particularly cooked vegetables prevalent in Chinese food culture, did not show significant associations with CVD risk in our study, aligning with findings suggesting modest benefits of vegetable intake on CVD outcomes [29, 44, 45]. This underscores the need for further research comparing the effects of raw versus cooked vegetable consumption in Eastern dietary contexts. While Western dietary patterns like DASH and Mediterranean diets have shown cardiovascular benefits, adapting these to regional dietary habits, such as the Chinese Heart-Healthy (CHH) diet, presents challenges in terms of palatability and adherence [15, 46, 47, 48, 49]. Therefore, future investigations should explore how different dietary frequencies and patterns influence CVD incidence, accounting for regional variations and cultural dietary practices.

Several studies have focused on developing and validating nomograms for CVD, utilizing various predictors such as coronary artery calcium scoring (CACS) and risk factors in asymptomatic adults, and prognostic models based on post-cardiac surgery nutritional status [50, 51]. However, the identification of dietary patterns related to CVD and the development of predictive models integrating multiple dietary factors remain crucial for secondary prevention strategies. Leveraging data from the baseline of the ChinaHEART study in Zhejiang province, which includes a large cohort of high-risk cardiac individuals, this study evaluates the impact of dietary intake frequencies on CVD incidence. The comprehensive dietary assessment, encompassing 12 food items, allowed us to analyze individual food types and their combined effects within the context of cardiovascular health. Methodologically, we employed rigorous statistical techniques including LASSO regression and multivariable Cox frailty models to prioritize and identify significant predictors associated with CVD, which minimized overfitting and enhanced the predictive accuracy of nomogram developed.

Despite these advances, this study still has several limitations. Firstly, dietary data were collected via questionnaires, potentially introducing recall and self-reporting reporting biases influenced by social desirability and portion size misunderstandings. This could lead to inaccuracies in reported food intake levels. Moreover, the food frequency questionnaires lacked quantitative measures of food consumption, limiting the precision of dietary analysis to comparisons of high versus low consumption levels. This lack of precision may lead to misclassification bias, where true dietary intake is not accurately reflected in the categories used, potentially diluting the observed associations between diet and CVD. The magnitude of this bias could be substantial, particularly if the actual intake levels vary widely within the broad categories used. Future studies should incorporate more precise dietary assessment methods and leverage comprehensive food composition databases to enhance accuracy [52, 53]. Secondly, internal validation was conducted using random samples within the dataset, necessitating external validation for broader clinical applicability. The study population was confined to the Zhejiang province, eastern China, highlighting the need for regional-specific predictive models due to variations in Chinese dietary patterns [54]. The direction of this bias is towards overestimating the model’s applicability and accuracy when applied to other populations. The magnitude of this bias could be large, as dietary habits and risk factors can vary significantly between regions. Ultimately, this study focused solely on stroke and CHD as CVD outcomes, excluding heart failure and other cardiovascular conditions. The direction of this bias is towards underestimating the overall impact of dietary factors on CVD, as other significant conditions like heart failure are not considered. Future research should encompass a broader spectrum of CVD outcomes to provide a comprehensive understanding of dietary impacts. Furthermore, the study’s low incidence rate of specific CVD events may introduce bias, underscoring the importance of extended follow-up periods and comprehensive outcome capture to refine predictive models aimed at dietary interventions for specific CVD outcomes [55]. This bias tends to underestimate the true association between dietary factors and CVD incidence due to insufficient statistical power to detect significant effects. The magnitude of this bias can be significant, particularly for rare outcomes, leading to potentially conservative estimates of dietary impacts. In conclusion, while this study offers valuable insights into the potential benefits of increased fruit, egg, and seafood consumption in reducing CVD risk, future interventional studies are warranted to validate these findings. Additionally, broader considerations such as food security and the length of the supply chain should be integrated into future research efforts to elucidate their impact on CVD incidence [56, 57].

## 5. Conclusions

The results from this study demonstrated daily consumption of eggs, seafood, and 1–3 days a week consumption of fruit decreased the incident risk of CVD. The nomogram, generated by incorporating statistically significant risk factors including age, sex, smoking, location, hr, SBP, fruit, eggs, and seafood, might be able to provide theoretical guidance and practical significance. Further intervention studies are warranted to validate these findings and explore optimal dietary strategies for CVD prevention.

## Data Availability

The data that support the findings of this study are available from the corresponding author, Yu, upon reasonable request.

## Abbreviations

CVD, cardiovascular disease; CHD, coronary heart disease; BMI, body mass index; DBP, diastolic blood pressure; SBP, systolic blood pressure; BP, blood pressure; hr, heart ratio; TG, triglyceride; TC, total cholesterol; LDL, low-density lipoprotein; HDL, high-density lipoprotein; CI, confidence interval; IQR, interquartile ranges; HR, hazard ratio; C-index, Harrell’s concordance index; DASH, dietary approach to stop hypertension; CACS, coronary artery calcium scoring; AUC, area under the ROC curves; ROC, receiver operator characteristic; RCT, randomized clinical trial.

## Supplementary Material

Supplementary material associated with this article can be found, in the online version, at https://doi.org/10.31083/j.rcm2511412.
