# Supplementary material for: Associations between Multiple Food Consumption Frequencies and the Incidence of Cardiovascular Disease in High Cardiac Risk Subjects
Source: Rev Cardiovasc Med. 2024 Nov 20;25(11):412. doi: 10.31083/j.rcm2511412 (PMC11607513; doi:10.31083/j.rcm2511412)
Supplement: Supplementary file 1 [file 2153-8174-25-11-412-s1.docx]

**List of Supplementary Information**

**Supplementary Table 1** Characteristics of food consumption frequency of high cardiac risk subjects in the training cohort and validation cohort.

**Supplementary Table 2** LASSO coefficients of 26 Variables at the minimum error.

**Supplementary Table 3** Univariate Cox and multivariate Cox regression model based on independent non-food variables screened by LASSO method.

**Supplementary Table 4** Association between consumption of various foods and the incidence of major cardiovascular diseases in high cardiac risk subjects in training cohort (n= 19,320).

**Supplementary Fig. 1** The decision curves analysis curve of the nomogram.

**Supplementary Table 1** Characteristics of food consumption frequency of high cardiac risk subjects in the training cohort and validation cohort.

|  | Training cohort  (No CVD) | Training cohort (CVD) | Validation cohort (No CVD) | Validation cohort (CVD) | p |  |
| --- | --- | --- | --- | --- | --- | --- |
| Rice (%) |  |  |  |  | 0.049 |  |
| 1 | 16772 (90.48) | 688 (87.87) | 8406 (90.90) | 357 (86.86) |  |  |
| 2 | 1066 (5.75) | 57 (7.28) | 524 (5.67) | 31 (7.54) |  |  |
| 3 | 519 (2.80) | 29 (3.70) | 239 (2.58) | 17 (4.14) |  |  |
| 4 | 98 (0.53) | 4 (0.51) | 42 (0.45) | 1 (0.24) |  |  |
| 5 | 82 (0.44) | 5 (0.64) | 37 (0.40) | 5 (1.22) |  |  |
| Wheat (%) |  |  |  |  | 0.546 |  |
| 1 | 993 (5.36) | 51 (6.51) | 483 (5.22) | 17 (4.14) |  |  |
| 2 | 2944 (15.88) | 137 (17.50) | 1494 (16.15) | 82 (19.95) |  |  |
| 3 | 7649 (41.26) | 310 (39.59) | 3794 (41.03) | 168 (40.88) |  |  |
| 4 | 4927 (26.58) | 200 (25.54) | 2456 (26.56) | 106 (25.79) |  |  |
| 5 | 2024 (10.92) | 85 (10.86) | 1021 (11.04) | 38 (9.25) |  |  |
| Grain (%) |  |  |  |  | 0.822 |  |
| 1 | 454 (2.45) | 24 (3.07) | 205 (2.22) | 9 (2.19) |  |  |
| 2 | 1079 (5.82) | 49 (6.26) | 555 (6.00) | 24 (5.84) |  |  |
| 3 | 6440 (34.74) | 269 (34.36) | 3195 (34.55) | 137 (33.33) |  |  |
| 4 | 6958 (37.54) | 294 (37.55) | 3523 (38.09) | 171 (41.61) |  |  |
| 5 | 3606 (19.45) | 147 (18.77) | 1770 (19.14) | 70 (17.03) |  |  |
| Meat (%) |  |  |  |  | 0.069 |  |
| 1 | 1463 (7.89) | 54 (6.90) | 765 (8.27) | 34 (8.27) |  |  |
| 2 | 4230 (22.82) | 164 (20.95) | 2130 (23.03) | 76 (18.49) |  |  |
| 3 | 8172 (44.08) | 344 (43.93) | 4135 (44.71) | 192 (46.72) |  |  |
| 4 | 3542 (19.11) | 168 (21.46) | 1730 (18.71) | 84 (20.44) |  |  |
| 5 | 1130 (6.10) | 53 (6.77) | 488 (5.28) | 25 (6.08) |  |  |
| Poultry (%) |  |  |  |  | 0.013 |  |
| 1 | 669 (3.61) | 32 (4.09) | 366 (3.96) | 21 (5.11) |  |  |
| 2 | 2916 (15.73) | 123 (15.71) | 1505 (16.27) | 60 (14.60) |  |  |
| 3 | 4703 (25.37) | 195 (24.90) | 2318 (25.06) | 89 (21.65) |  |  |
| 4 | 5951 (32.10) | 216 (27.59) | 2964 (32.05) | 126 (30.66) |  |  |
| 5 | 4298 (23.19) | 217 (27.71) | 2095 (22.65) | 115 (27.98) |  |  |
| Seafood (%) |  |  |  |  | <0.001 |  |
| 1 | 1624 (8.76) | 40 (5.11) | 813 (8.79) | 35 (8.52) |  |  |
| 2 | 3372 (18.19) | 120 (15.33) | 1691 (18.29) | 63 (15.33) |  |  |
| 3 | 6158 (33.22) | 271 (34.61) | 3115 (33.68) | 118 (28.71) |  |  |
| 4 | 4961 (26.76) | 210 (26.82) | 2452 (26.51) | 114 (27.74) |  |  |
| 5 | 2422 (13.07) | 142 (18.14) | 1177 (12.73) | 81 (19.71) |  |  |
| Egg (%) |  |  |  |  | 0.007 |  |
| 1 | 238 (1.28) | 7 (0.89) | 93 (1.01) | 4 (0.97) |  |  |
| 2 | 1607 (8.67) | 51 (6.51) | 829 (8.96) | 27 (6.57) |  |  |
| 3 | 6651 (35.88) | 259 (33.08) | 3313 (35.82) | 134 (32.60) |  |  |
| 4 | 7323 (39.50) | 325 (41.51) | 3667 (39.65) | 167 (40.63) |  |  |
| 5 | 2718 (14.66) | 141 (18.01) | 1346 (14.55) | 79 (19.22) |  |  |
| Vegetable (%) |  |  |  |  | 0.192 |  |
| 1 | 15793 (85.20) | 669 (85.44) | 7987 (86.36) | 353 (85.89) |  |  |
| 2 | 1370 (7.39) | 57 (7.28) | 609 (6.59) | 27 (6.57) |  |  |
| 3 | 715 (3.86) | 35 (4.47) | 350 (3.78) | 11 (2.68) |  |  |
| 4 | 580 (3.13) | 18 (2.30) | 273 (2.95) | 18 (4.38) |  |  |
| 5 | 79 (0.43) | 4 (0.51) | 29 (0.31) | 2 (0.49) |  |  |
| Pickle (%) |  |  |  |  | 0.025 |  |
| 1 | 151 (0.81) | 4 (0.51) | 66 (0.71) | 1 (0.24) |  |  |
| 2 | 1198 (6.46) | 35 (4.47) | 653 (7.06) | 17 (4.14) |  |  |
| 3 | 6098 (32.90) | 243 (31.03) | 3069 (33.19) | 126 (30.66) |  |  |
| 4 | 7538 (40.66) | 335 (42.78) | 3714 (40.16) | 187 (45.50) |  |  |
| 5 | 3552 (19.16) | 166 (21.20) | 1746 (18.88) | 80 (19.46) |  |  |
| Fruit (%) |  |  |  |  | <0.001 |  |
| 1 | 1365 (7.36) | 58 (7.41) | 702 (7.59) | 29 (7.06) |  |  |
| 2 | 1985 (10.71) | 66 (8.43) | 1019 (11.02) | 30 (7.30) |  |  |
| 3 | 6193 (33.41) | 225 (28.74) | 2984 (32.27) | 134 (32.60) |  |  |
| 4 | 5641 (30.43) | 232 (29.63) | 2779 (30.05) | 126 (30.66) |  |  |
| 5 | 3353 (18.09) | 202 (25.80) | 1764 (19.07) | 92 (22.38) |  |  |
| Bean (%) |  |  |  |  | 0.374 |  |
| 1 | 241 (1.30) | 15 (1.92) | 107 (1.16) | 5 (1.22) |  |  |
| 2 | 1764 (9.52) | 70 (8.94) | 887 (9.59) | 25 (6.08) |  |  |
| 3 | 7022 (37.88) | 281 (35.89) | 3480 (37.63) | 158 (38.44) |  |  |
| 4 | 6787 (36.61) | 302 (38.57) | 3405 (36.82) | 151 (36.74) |  |  |
| 5 | 2723 (14.69) | 115 (14.69) | 1369 (14.80) | 72 (17.52) |  |  |
| Milk (%) |  |  |  |  | 0.294 |  |
| 1 | 164 (0.88) | 8 (1.02) | 87 (0.94) | 6 (1.46) |  |  |
| 2 | 477 (2.57) | 13 (1.66) | 271 (2.93) | 12 (2.92) |  |  |
| 3 | 3609 (19.47) | 151 (19.28) | 1775 (19.19) | 75 (18.25) |  |  |
| 4 | 4537 (24.48) | 175 (22.35) | 2213 (23.93) | 113 (27.49) |  |  |
| 5 | 9750 (52.60) | 436 (55.68) | 4902 (53.01) | 205 (49.88) |  |  |

Food consumption frequency: 1= Daily; 2= 4-6 days a week; 3= 1-3 days a week; 4= 1-3 days a month; 5= None or little.

**Supplementary Table 2** LASSO coefficients of 26 Variables at the minimum error.

| Variables | Coefficient |
| --- | --- |
| Age | 0.084 |
| Sex | -0.41 |
| BMI | 0.011 |
| SBP | 0.0089 |
| DBP | 0.0057 |
| HR | 0.0071 |
| TC | 0.057 |
| HDL | -0.077 |
| TG | 0 |
| LDL | -0.05 |
| Location | 0.41 |
| Alcohol | -0.11 |
| Education | -0.056 |
| Smoke | 0.28 |
| Rice | 0.088 |
| Wheat | -0.057 |
| Grain | 0 |
| Meat | 0.038 |
| Poultry | -0.085 |
| Seafood | 0.061 |
| Egg | 0.14 |
| Vegetable | 0 |
| Pickle | 0.046 |
| Fruit | 0.099 |
| Bean | -0.037 |
| Dairy | 0.024 |

Abbreviations: BMI: body mass index, SBP: systolic blood pressure, DBP: diastolic blood pressure, HR: heart ratio, TC: total cholesterol, TG: triglyceride, HDL: high-density lipoprotein, LDL: low-density lipoprotein, LASSO: Least absolute shrinkage and selection operator.

**Supplementary Table 3** Univariate Cox and multivariate Cox regression model based on independent non-food variables screened by LASSO method.

| Variables | Univariate Cox | | Multivariate Cox | |
| --- | --- | --- | --- | --- |
|  | HR (95%CI) | *p* value | HR (95%CI) | *p* value |
| Sex |  |  |  |  |
| Male | Ref | - | Ref | - |
| Female | 0.61 (0.53-0.7) | <0.05 | 0.66 (0.55-0.8) | <0.05 |
| Age | 1.09 (1.08-1.10) | <0.05 | 1.09 (1.08-1.10) | <0.05 |
| LDL | 0.90 (0.85-0.96) | <0.05 | 0.92 (0.82-1.03) | 0.16 |
| HDL | 1.10 (0.95-1.27) | 0.20 | 0.92 (0.78-1.1) | 0.38 |
| TC | 0.95 (0.9-1) | 0.074 | 1.08 (0.98-1.19) | 0.11 |
| HR | 1.01 (1-1.01) | 0.069 | 1.01 (1-1.01) | <0.05 |
| DBP | 1.00 (1-1.01) | 0.16 | 1.01 (1-1.01) | 0.069 |
| SBP | 1.02 (1.01-1.02) | <0.05 | 1.01 (1-1.01) | <0.05 |
| BMI | 1.00 (0.98-1.02) | 0.88 | 1.01 (0.99-1.04) | 0.30 |
| Smoke |  |  |  |  |
| 0 | Ref | - | Ref | - |
| 1 | 1.51 (1.29-1.77) | <0.05 | 1.36 (1.13-1.64) | <0.05 |
| Alcohol |  |  |  |  |
| 0 | Ref | - | Ref | - |
| 1 | 1.18 (1.00-1.39) | 0.055 | 0.89 (0.74-1.07) | 0.21 |
| Location |  |  |  |  |
| Rural | Ref | - | Ref | - |
| Urban | 1.29 (0.88-1.90) | 0.19 | 1.57 (1.07-2.29) | <0.05 |
| Education |  |  |  |  |
| Post-secondary school | Ref | - | Ref | - |
| Pre-secondary school | 2.01 (1.38-2.92) | <0.05 | 1.11 (0.75-1.63) | 0.60 |
| Secondary school | 1.41 (0.98-2.02) | 0.063 | 0.95 (0.66-1.36) | 0.77 |

Abbreviations: BMI: body mass index, SBP: systolic blood pressure, DBP: diastolic blood pressure, HR: heart ratio, TC: total cholesterol, TG: triglyceride, HDL: high-density lipoprotein, LDL: low-density lipoprotein.

**Supplementary Table 4** Association between consumption of various foods and the incidence of major cardiovascular diseases in high cardiac risk subjects in training cohort (n= 19,320).

| Food category | Daily | 4-6 days a week | 1-3 days a week | 1-3 days a month | None or little | *P* trend |
| --- | --- | --- | --- | --- | --- | --- |
| Rice (Major cardiovascular disease events) | 688 | 57 | 29 | 4 | 5 |  |
| Rice (%) | 3.94 | 5.08 | 5.29 | 3.92 | 5.75 |  |
| Rice (model1) | Ref | 1.19 (0.88-1.59) | 1.40 (0.95-2.06) | 1.09 (0.41-2.95) | 1.20 (0.49-2.92) | 0.098 |
| Rice (model2) | Ref | 1.15 (0.86-1.55) | 1.34 (0.91-1.97) | 1.04 (0.39-2.82) | 1.12 (0.46-2.74) | 0.18 |
| Wheat (Major cardiovascular disease events) | 51 | 137 | 310 | 200 | 85 |  |
| Wheat (%) | 4.89 | 4.45 | 3.89 | 3.9 | 4.03 |  |
| Wheat (model1) | Ref | 0.88 (0.63-1.22) | 0.81 (0.60-1.10) | 0.81 (0.59-1.13) | 0.95 (0.66-1.39) | 0.67 |
| Wheat (model2) | Ref | 0.87 (0.62-1.20) | 0.81 (0.60-1.09) | 0.80 (0.58-1.11) | 0.90 (0.62-1.31) | 0.53 |
| Grain (Major cardiovascular disease events) | 24 | 49 | 269 | 294 | 147 |  |
| Grain (%) | 5.02 | 4.34 | 4.01 | 4.05 | 3.92 |  |
| Grain (model1) | Ref | 0.85 (0.52-1.39) | 0.82 (0.53-1.25) | 0.80 (0.52-1.23) | 0.91 (0.58-1.43) | 0.88 |
| Grain (model2) | Ref | 0.82 (0.50-1.34) | 0.79 (0.51-1.21) | 0.78 (0.51-1.20) | 0.88 (0.56-1.37) | 0.94 |
| Meat (Major cardiovascular disease events) | 54 | 164 | 344 | 168 | 53 |  |
| Meat (%) | 3.56 | 3.73 | 4.04 | 4.53 | 4.48 |  |
| Meat (model1) | Ref | 1.18 (0.86-1.61) | 1.22 (0.91-1.63) | 1.32 (0.96-1.81) | 1.30 (0.88-1.92) | 0.10 |
| Meat (model2) | Ref | 1.21 (0.89-1.66) | 1.26 (0.94-1.69) | 1.36 (0.99-1.87) | 1.32 (0.89-1.94) | 0.086 |
| Poultry (Major cardiovascular disease events) | 32 | 123 | 195 | 216 | 217 |  |
| Poultry (%) | 4.56 | 4.05 | 3.98 | 3.5 | 4.81 |  |
| Poultry (model1) | Ref | 1.09 (0.73-1.63) | 1.16 (0.79-1.71) | 0.96 (0.65-1.43) | 1.24 (0.83-1.85) | 0.34 |
| Poultry (model2) | Ref | 1.12 (0.75-1.66) | 1.18 (0.80-1.73) | 0.99 (0.66-1.48) | 1.25 (0.84-1.86) | 0.35 |
| Seafood (Major cardiovascular disease events) | 40 | 120 | 271 | 210 | 142 |  |
| Seafood (%) | 2.4 | 3.44 | 4.22 | 4.06 | 5.54 |  |
| Seafood (model1) | Ref | 1.43 (0.97-2.10) | 1.75 (1.20-2.55) | 1.62 (1.09-2.40) | 1.97 (1.31-2.96) | 0.0059 |
| Seafood (model2) | Ref | 1.44 (0.98-2.12) | 1.72 (1.18-2.51) | 1.59 (1.07-2.35) | 1.86 (1.24-2.81) | 0.024 |
| Egg (Major cardiovascular disease events) | 7 | 51 | 259 | 325 | 141 |  |
| Egg (%) | 2.86 | 3.08 | 3.75 | 4.25 | 4.93 |  |
| Egg (model1) | Ref | 1.27 (0.58-2.82) | 1.37 (0.65-2.91) | 1.58 (0.75-3.35) | 1.74 (0.81-3.74) | 0.0097 |
| Egg (model2) | Ref | 1.23 (0.56-2.72) | 1.32 (0.62-2.80) | 1.55 (0.73-3.29) | 1.70 (0.79-3.64) | 0.0073 |
| Vegetable (Major cardiovascular disease events) | 669 | 57 | 35 | 18 | 4 |  |
| Vegetable (%) | 4.06 | 3.99 | 4.67 | 3.01 | 4.82 |  |
| Vegetable (model1) | Ref | 1.15 (0.86-1.53) | 1.39 (0.98-1.97) | 0.71 (0.44-1.14) | 1.35 (0.50-3.63) | 0.80 |
| Vegetable (model2) | Ref | 1.13 (0.85-1.51) | 1.39 (0.98-1.97) | 0.71 (0.44-1.14) | 1.40 (0.52-3.77) | 0.81 |
| Pickle (Major cardiovascular disease events) | 4 | 35 | 243 | 335 | 166 |  |
| Pickle (%) | 2.58 | 2.84 | 3.83 | 4.26 | 4.46 |  |
| Pickle (model1) | Ref | 1.42 (0.50-4.02) | 1.49 (0.55-4.01) | 1.54 (0.57-4.13) | 1.58 (0.58-4.26) | 0.37 |
| Pickle (model2) | Ref | 1.39 (0.49-3.95) | 1.49 (0.55-4.01) | 1.53 (0.57-4.12) | 1.58 (0.58-4.26) | 0.36 |
| Fruit (Major cardiovascular disease events) | 58 | 66 | 225 | 232 | 202 |  |
| Fruit (%) | 4.08 | 3.22 | 3.51 | 3.95 | 5.68 |  |
| Fruit (model1) | Ref | 1.03 (0.72-1.49) | 0.91 (0.68-1.23) | 1.03 (0.76-1.40) | 1.33 (0.97-1.84) | 0.027 |
| Fruit (model2) | Ref | 1.04 (0.72-1.50) | 0.93 (0.69-1.25) | 1.06 (0.78-1.44) | 1.33 (0.96-1.84) | 0.027 |
| Bean (Major cardiovascular disease events) | 15 | 70 | 281 | 302 | 115 |  |
| Bean (%) | 5.86 | 3.82 | 3.85 | 4.26 | 4.05 |  |
| Bean (model1) | Ref | 0.70 (0.40-1.23) | 0.64 (0.38-1.07) | 0.68 (0.40-1.14) | 0.66 (0.38-1.15) | 0.68 |
| Bean (model2) | Ref | 0.67 (0.38-1.18) | 0.61 (0.36-1.03) | 0.65 (0.38-1.10) | 0.63 (0.36-1.09) | 0.62 |
| Dairy (Major cardiovascular disease events) | 8 | 13 | 151 | 175 | 436 |  |
| Dairy (%) | 4.65 | 2.65 | 4.02 | 3.71 | 4.28 |  |
| Dairy (model1) | Ref | 0.87 (0.36-2.11) | 1.02 (0.50-2.08) | 0.96 (0.47-1.96) | 1.10 (0.55-2.23) | 0.24 |
| Dairy (model2) | Ref | 0.89 (0.37-2.16) | 1.01 (0.50-2.07) | 0.98 (0.48-1.99) | 1.10 (0.54-2.22) | 0.28 |

Abbreviations: CVD events = Major cardiovascular disease events (coronary heart disease and stroke). Ref = Reference group, HR = Hazard Ratio, CI = Confidence Interval, BMI: body mass index, SBP: systolic blood pressure, DBP: diastolic blood pressure, HR: heart ratio, TC: total cholesterol, TG: triglyceride, HDL: high-density lipoprotein, LDL: low-density lipoprotein. Model 1 adjusted for age, sex, and center was included as a random effect. Model 2 adjusted for age, sex, alcohol, location, smoke, education, BMI, LDL, HDL, TC, HR, DBP, SBP, and center was also included as a random effect.


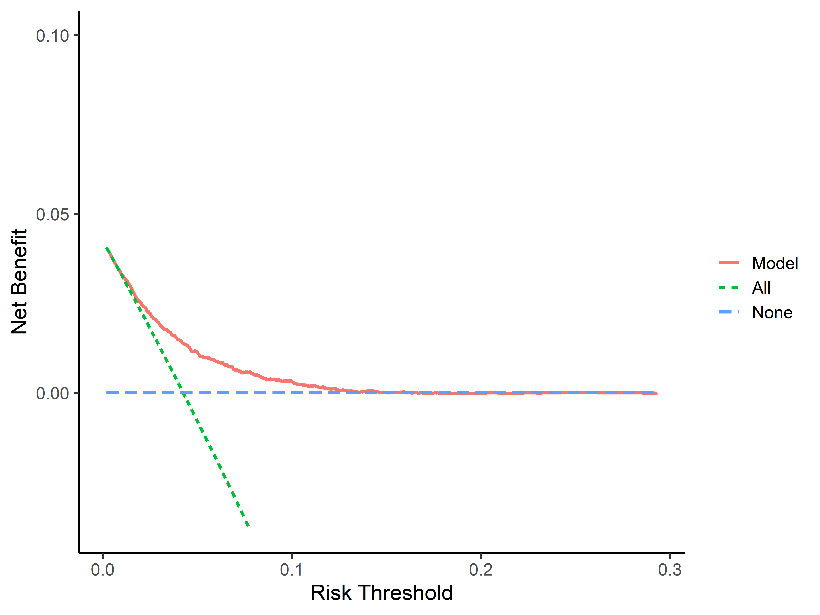


**Supplementary Fig. 1** The decision curves analysis curve of the nomogram.

Abbreviations: DCA: decision curves analysis. The net benefit of the model diagnosis will be larger when the closer the curve is to the top of the DCA graph.
